# Supplementary material for: Genomic analysis and clinical correlations of non-small cell lung cancer brain metastasis
Source: Nat Commun. 2023 Aug 17;14:4980. doi: 10.1038/s41467-023-40793-x (PMC10435547; doi:10.1038/s41467-023-40793-x)
Supplement: Supplementary file 1 — Supplementary Information [file 41467_2023_40793_MOESM1_ESM.pdf]

Supplemental Figure 1

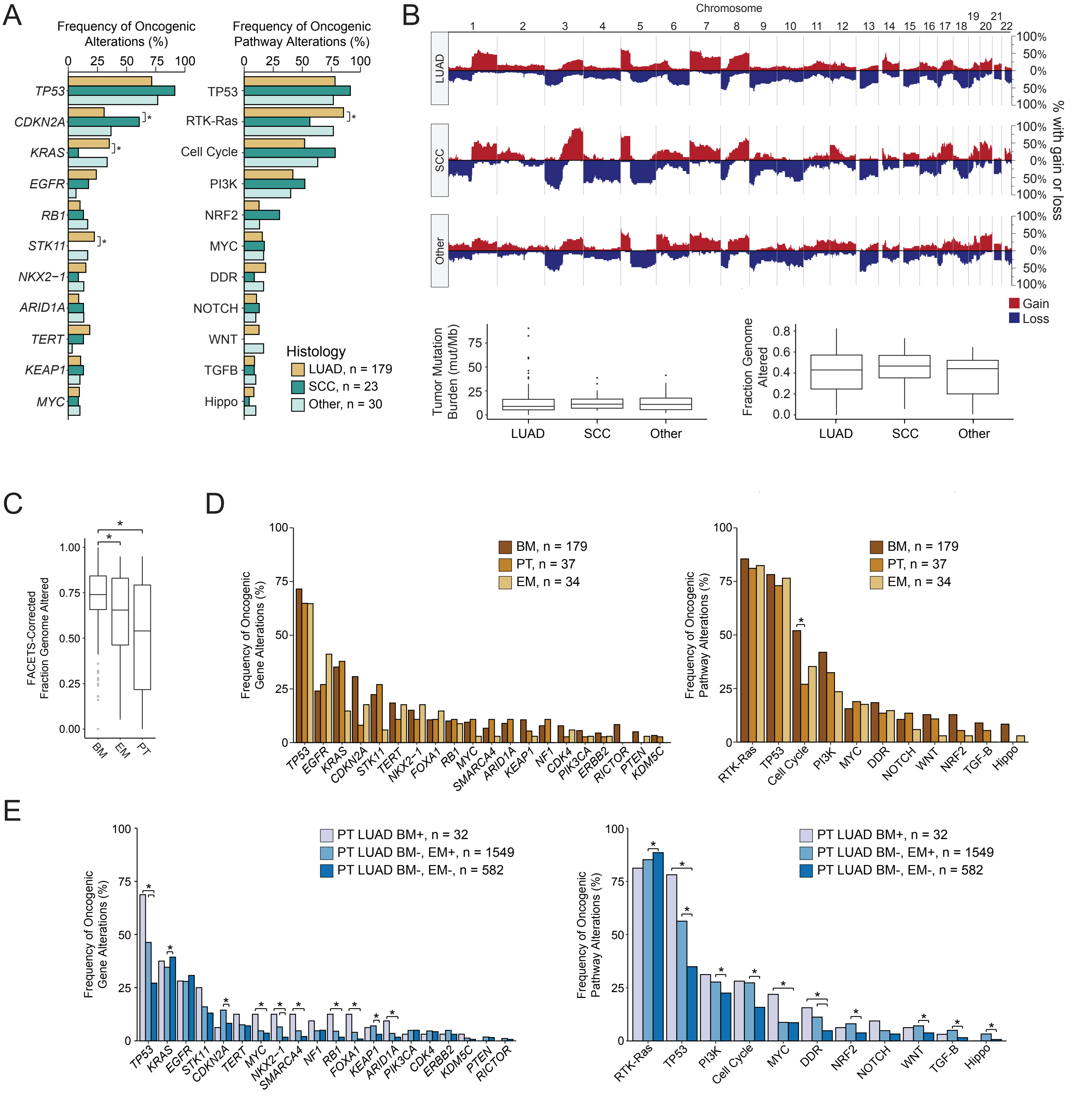

Supplemental Figure 1. Genomic differences of BM NSCLC based on different histologic types and frequency of genomic alterations in primary tumors with different metastatic disease status. A, Comparison of the oncogenic alterations at both the gene and pathway level between BM samples of different histologic subtypes (LUAD – lung adenocarcinoma, SCC – squamous cell carcinoma, other – other NSCLC histologies). We found an enrichment of KRAS and STK11 alterations (*KRAS*: 35% vs 9%, p = 0.009, q = 0.049; *STK11*: 22% vs 0%, p = 0.01, q = 0.049), as well as RTK-Ras pathway alterations in LUAD BM samples compared to SCC BM samples (86% vs 57%, p = 0.002, q = 0.022. B, Comparison of the genome-wide copy number profiles for LUAD (n = 179), SCC (n = 23), and other (n = 30) histologic subtypes shown on top, and a comparison of the tumor mutation burden (TMB) and fraction genome altered (FGA) by histologic subtype shown on the bottom. C, Purity-corrected fraction genome altered comparison between BM, EM, and PT samples. BM samples had significantly higher FACETS-corrected FGA estimates compared to PT (p = 0.00011) and EM (p = 0.01632). D, Comparison of oncogenic alterations between BM, PT, and EM samples, restricting only to LUAD samples. The cell cycle pathway was significantly enriched in BM tumors compared to PT tumors (p = 0.007, q = 0.072). E, Comparison of oncogenic alterations between primary LUAD samples from patients with BM (PT LUAD BM+), primary LUAD samples from metastatic patients without BM (PT LUAD BM-, EM+), and primary LUAD samples from non-metastatic patients (PT LUAD BM-, EM-). The following genes were significantly enriched after p-value correction in PT LUAD BM+ vs PT LUAD BM-, EM- tumors: *TP53*, q = 5.09e-5; *MYC*, q = 0.083; *NKX2-1*, q = 0.022; *SMARCA4*, q = 0.0299; *RB1*, q = 0.0221; *FOXA1*, q = 0.007; *ARID1A*, q = 0.068. The following genes were significantly enriched in PT LUAD BM-, EM+ vs PT LUAD BM-, EM- tumors: *TP53*, q = 5.09e-5; *KRAS*, q = 0.086; *CDKN2A*, q = 2.42e-4; *NKX2-1*, q = 1.00e-5; *KEAP1*, q = 1.03e-3.

Statistical significance for all comparisons of categorical variables was performed using a two-sided Fisher's exact test. Multiple hypotheses testing was performed using a Benjamini-Hochberg correction. All continuous features were compared using a two-sided Mann–Whitney U-test. The center line of the box plots indicates the median. The bounds of the box indicate the interquartile range. The whiskers indicate the highest and lowest values not considered outliers. Asterisks indicates significance between groups being compared.

Source data are provided as a Source Data file for Supplemental Figure 1.

Supplemental Figure 2

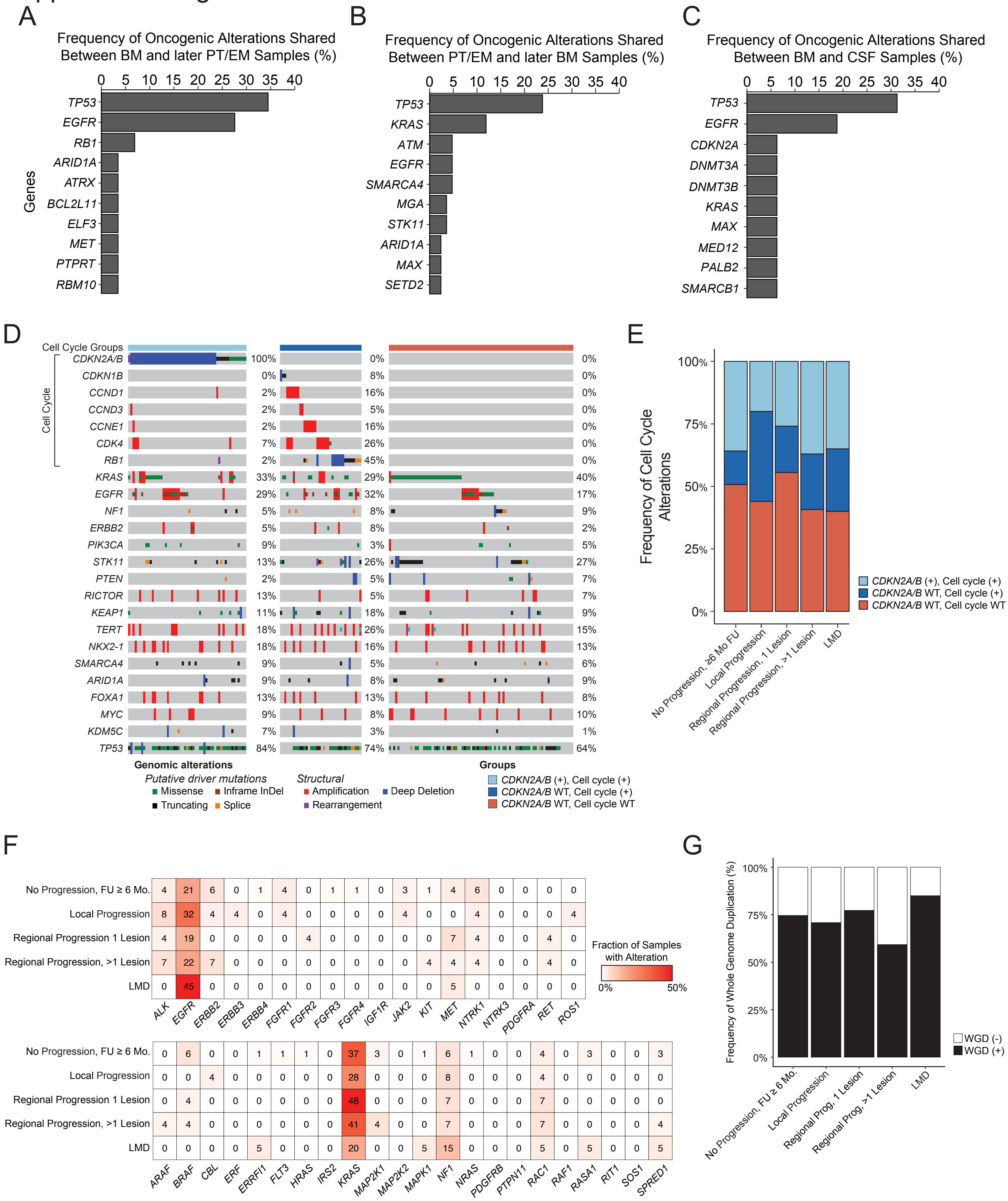

Supplemental Figure 2. Genomic characterization of disease progression groups. A, Oncogenic mutations that were shared between BM and PT/EM tumor samples when the PT/EM occurred later. B, Oncogenic mutations that were shared between BM and PT/EM tumor samples when the BM sampling occurred later. C, Oncogenic mutations that were shared between BM and CSF samples. D, Oncoprint depicting different cell cycle groups in BM LUAD cohort: *CDKN2A/B* and cellcycle positive, *CDKN2A/B* wild type (WT) and cell cycle positive, and both *CDKN2A/B* and cell cycle WT. E, Frequency of cell cycle alterations among different disease progression groups. F, Heatmap of the oncogenic alteration frequencies within the RTK-RAS pathway, stratified by the type of intracranial progression. G, Frequency of whole-genome duplication (WGD) stratified by the type of intracranial progression. Source data are provided as a Source Data file for Supplemental Figure 2.

Supplemental Table 1: Patient and Treatment Characteristics of Lung Adenocarcinoma Cohort

**Patient Characteristics**	**Total 179, N (%)**
**Sex, No. (%)**	
Female	113 (63)
Male	66 (37)
**Smoking Status, No. (%)**	
Current	38 (21)
Former	101 (56)
Never	40 (23)
**Age**, Median (range)	67 (31-91)
**KPS**, Median (range)	80 (40-100)
**Number of BM at Resection**, No. (%)	
1	93 (52)
2-5	60 (33)
6-15	25 (14)
>15	1 (1)
**Diameter of Largest Brain Metastasis**, **cm** Median (range)	2.9 (0.9 - 7.0)
**Neurologic Symptoms at Resection,** No. (%)	
Yes	164 (92)
No	15 (8)
**Treatment Prior to Resection**	
**None**, No. (%)	97 (54)
**Systemic therapy*, No. (%)**	81 (45)
Cytotoxic chemotherapy	50 (62)
Immunotherapy	14 (17)
Tyrosine Kinase Inhibitor	12 (15)
VEGF Inhibitor	3 (4)
Other	2 (2)
**Radiation Therapy**, No. (%)	11 (6)
Stereotactic Radiosurgery	8 (73)
Whole-Brain Radiotherapy	2 (18)
Prophylactic Cranial Irradiation	1 (9)

*Received either monotherapy or combination therapy as the most recent therapy prior to resection
