# Supplementary material for: Genomic analysis and clinical correlations of non-small cell lung cancer brain metastasis
Source: Nat Commun. 2023 Aug 17;14:4980. doi: 10.1038/s41467-023-40793-x (PMC10435547; doi:10.1038/s41467-023-40793-x)
Supplement: Supplementary file 3 — Description of Additional Supplementary Files [file 41467_2023_40793_MOESM3_ESM.pdf]

Description of Additional Supplementary Files

Supplementary Data 1: Clinical data for 233 brain metastasis patients.

Supplementary Data 2: Mutation annotation file for all mutations for samples included in analysis.

Supplementary Data 3: Gene-level binary matrix for all oncogenic alterations.

Supplementary Data 4: Pathway-level binary matrix for all oncogenic pathway alterations.
