# Supplementary material for: Genomic analysis and clinical correlations of non-small cell lung cancer brain metastasis
Source: Nat Commun. 2023 Aug 17;14:4980. doi: 10.1038/s41467-023-40793-x (PMC10435547; doi:10.1038/s41467-023-40793-x)
Supplement: Supplementary file 8 — Reporting Summary [file 41467_2023_40793_MOESM8_ESM.pdf]

nature portfolio

Corresponding author(s): Pike, Luke R.

Last updated by author(s): Aug 2, 2023

Reporting Summary

Nature Portfolio wishes to improve the reproducibility of the work that we publish. This form provides structure for consistency and transparency in reporting. For further information on Nature Portfolio policies, see our Editorial Policies and the Editorial Policy Checklist.

Statistics

For all statistical analyses, confirm that the following items are present in the figure legend, table legend, main text, or Methods section.

n/a	Confirmed	
☐	☒	The exact sample size (*n*) for each experimental group/condition, given as a discrete number and unit of measurement
☐	☒	A statement on whether measurements were taken from distinct samples or whether the same sample was measured repeatedly
☐	☒	The statistical test(s) used AND whether they are one- or two-sided<br>*Only common tests should be described solely by name; describe more complex techniques in the Methods section.*
☐	☒	A description of all covariates tested
☐	☒	A description of any assumptions or corrections, such as tests of normality and adjustment for multiple comparisons
☐	☒	A full description of the statistical parameters including central tendency (e.g. means) or other basic estimates (e.g. regression coefficient) AND variation (e.g. standard deviation) or associated estimates of uncertainty (e.g. confidence intervals)
☐	☒	For null hypothesis testing, the test statistic (e.g. *F*, *t*, *r*) with confidence intervals, effect sizes, degrees of freedom and *P* value noted<br>*Give P values as exact values whenever suitable.*
☒	☐	For Bayesian analysis, information on the choice of priors and Markov chain Monte Carlo settings
☐	☒	For hierarchical and complex designs, identification of the appropriate level for tests and full reporting of outcomes
☒	☐	Estimates of effect sizes (e.g. Cohen's *d*, Pearson's *r*), indicating how they were calculated

Our web collection on statistics for biologists contains articles on many of the points above.

Software and code

Policy information about availability of computer code

Data collection	Data was collected using retrospective chart review of consented and eligible patients. Progression events were reviewed and confirmed by senior radiation oncology resident and radiation oncology attending independently.
Data analysis	No custom code was developed for this project. FACETS, an allele-specific copy-number algorithm, was used to generate purity-corrected estimates of the fraction of genome altered for patients. Versions used were: FACETS version: 0.6.2, FACETS-suite version: 2.0.8.

For manuscripts utilizing custom algorithms or software that are central to the research but not yet described in published literature, software must be made available to editors and reviewers. We strongly encourage code deposition in a community repository (e.g. GitHub). See the Nature Portfolio guidelines for submitting code & software for further information.

Data

Policy information about availability of data

All manuscripts must include a data availability statement. This statement should provide the following information, where applicable:
- Accession codes, unique identifiers, or web links for publicly available datasets
- A description of any restrictions on data availability
- For clinical datasets or third party data, please ensure that the statement adheres to our policy

The raw sequencing data for the MSK-IMPACT analysis is protected and cannot be broadly available due to privacy laws; patient consent to deposit raw sequencing

data was not obtained. De-identified data are available under restricted access to protect patient privacy in accordance with federal and state law. Raw data may be requested from schultzn@mskcc.org with appropriate institutional approvals. Data will be shared for a span of 2 years within 2 weeks of execution of a data transfer agreement with MSK, which will retain all title and rights to the data and results from their use. All de-identified clinical and genomic data for the patients in this study have been deposited in the cBioPortal for Cancer Genomics9,30 and are publicly available for browsing and download at https://www.cbioportal.org/study/summary?id=bm_nsclc_mskcc_2023. Source data are provided with this paper.

Human research participants

Policy information about studies involving human research participants and Sex and Gender in Research.

Reporting on sex and gender	Participant's sex is reported in Table 1.
Population characteristics	Population characteristics are summarized in Table 1.
Recruitment	Patient selection criteria included diagnosis of NSCLC and SOC craniotomy for BM resection during the period of time from January 2010 until April 2021 with samples undergoing sequencing by one of the versions of Memorial Sloan Kettering-Integrated Molecular Profiling of Actionable Cancer Targets (MSK-IMPACT) assay (341, 410, 468, 505). We were able to identify 249 patients. We then went ahead and collected information about clinical course of patients that included baseline patient characteristics, prior systemic therapy, radiotherapy (RT), and CNS-specific clinical outcomes, including local control, regional control, and development of leptomeningeal disease (LMD). This yielded cohort of 233 patients. Patient and treatment characteristics are summarized in Table 1.
Ethics oversight	The study was approved by the MSK Institutional Review and Privacy Board.

Note that full information on the approval of the study protocol must also be provided in the manuscript.

Field-specific reporting

Please select the one below that is the best fit for your research. If you are not sure, read the appropriate sections before making your selection.

☒ Life sciences ☐ Behavioural & social sciences ☐ Ecological, evolutionary & environmental sciences

For a reference copy of the document with all sections, see nature.com/documents/nr-reporting-summary-flat.pdf

Life sciences study design

All studies must disclose on these points even when the disclosure is negative.

Sample size	Samples were identified after submitting an institutional data extraction request with following eligibility criteria: NSCLC histology, standard of care craniotomy and MSK-IMPACT available for all resected BM samples. We initially identified 249 samples, but 16 samples were excluded due to various reasons such as clinical data limitation, lack of continuous follow up. All genomic data will be made available upon publication.
Data exclusions	Absence of clinical follow up and lack of information about CNS-specific outcomes (excluded 15 samples). 1 sample was excluded due to enriched MSI.
Replication	Replication was not possible due to limited number of identified samples.
Randomization	The absence of randomization in this study was primarily attributed to its retrospective nature, which posed practical limitations on implementing random assignment of participants to different groups.
Blinding	The retrospective nature of this study, coupled with its non-clinical trial design, led to the decision of not implementing blinding

Reporting for specific materials, systems and methods

We require information from authors about some types of materials, experimental systems and methods used in many studies. Here, indicate whether each material, system or method listed is relevant to your study. If you are not sure if a list item applies to your research, read the appropriate section before selecting a response.

Materials & experimental systems

n/a	Involved in the study
☒	☐ Antibodies
☒	☐ Eukaryotic cell lines
☒	☐ Palaeontology and archaeology
☒	☐ Animals and other organisms
☐	☒ Clinical data
☒	☐ Dual use research of concern

Methods

n/a	Involved in the study
☒	☐ ChIP-seq
☒	☐ Flow cytometry
☒	☐ MRI-based neuroimaging

Clinical data

Policy information about clinical studies

All manuscripts should comply with the ICMJE guidelines for publication of clinical research and a completed CONSORT checklist must be included with all submissions.

Clinical trial registration: Not applicable. No therapeutic clinical trial is reported.

Study protocol: Institutional sequencing protocol was used to analyze data from clinical sequencing (MSK IRB #12-245). Retrospective research protocol (MSK IRB#16-314) was used to collect clinical information

Data collection: Samples were initially identified retrospectively through a data query at MSKCC. Retrospective chart review was perfeormed and confirmed by a at least two senior investigators.

Outcomes: Pathology and radiology reports as well as medical oncology, surgery, and radiation oncology notes were reviewed in the electronic medical record to annotate clinical characteristics for patients with BM NSCLC. Outcomes included NSCLC histology, metastases description (size, quantity) , previous systemic and radiation therapy received, and CNS-specific clinical outcomes, including local control, regional control, and development of leptomeningeal disease (LMD).
